# Range of motion changes in upper and lower limb joints according to locomotion speed in healthy adults: A cross-sectional observational study

**DOI:** 10.1097/MD.0000000000048905

**Published:** 2026-05-29

**Authors:** Nam-Jin Jung, MinBong Kang

**Affiliations:** a Department of Physical Therapy, Taegu Science University, Daegu, Republic of Korea; b Department of Physical Therapy, Daegu Medical Foundation K Hospital, Daegu, Republic of Korea.

**Keywords:** gait speed, interlimb coordination, kinematics change, locomotion, range of motion

## Abstract

It is imperative to consider the coordination between the upper and lower extremities during locomotion, as this is a crucial aspect of achieving efficient locomotion. The present cross-sectional observational study aimed to analyze the three-dimensional kinematic characteristics of the upper and lower extremity joints in healthy adults as a function of locomotion speed. From September 2023 to March 2024, 155 healthy adults without neuromusculoskeletal disorders were enrolled in the study. The measurement of joint range of motion and angular velocity was conducted utilizing a 16-channel three-dimensional motion analysis system (iSen) under conditions of normal locomotion (100%), fast locomotion (130%), and running (150%). One-way repeated measures analysis of variance was performed to compare joint movements according to locomotion speed, with Bonferroni post hoc analysis (α = 0.0167). A total of 155 healthy adults (age: 24.3 ± 2.8 years; 82 males, 73 females) participated. A significant increase in joint range of motion was observed with increasing locomotion speed in all joints except pelvic transverse plane rotation (*P* < .05). Changes in the coordinated movements of the upper and lower extremities according to locomotion speed are thought to be an active strategy for locomotion stability and efficiency.

## 1. Introduction

Locomotion can be defined as the most fundamental human motor function, and is defined as an integrated movement pattern achieved through the delicate coordination of the body’s neuromusculoskeletal system.^[1]^ The occurrence of neurological and musculoskeletal disorders can be observed through abnormal locomotion patterns, and various types of locomotion analysis research are being conducted to quantitatively assess these locomotion disorders.^[2]^

Research in the field of locomotion analysis has historically centered on the locomotion cycle and the biomechanical characteristics of the lower extremities.^[3,4]^ However, recent studies have shifted their focus to the impact of active upper extremity movement on locomotion efficiency and overall motor coordination during movement.^[5,6]^ This paradigm shift in research is predicated on the recognition that the upper extremities are not merely passive structures that follow the locomotion, but rather a pivotal element that actively contributes to overall locomotion stability and efficiency.^[7]^

During normal locomotion, the upper limbs exhibit a reciprocal movement pattern, moving in the opposite direction to the lower limbs.^[7]^ This movement is theorized to counteract the angular momentum generated in the lower limbs during locomotion, thereby enhancing the overall efficiency of movement and positively influencing balance.^[6]^

The movement of the upper limbs that occurs in an alternating pattern has been explained from various perspectives in many studies. In the study by Huang et al,^[8]^ a novel movement pattern was identified that facilitates trunk and pelvis movement in patients with low back pain through alterations in arm swing. The investigation further revealed a significant correlation between the upper limbs, trunk, and pelvis during locomotion from a biomechanical standpoint. Moreover, a systematic literature review by Meyns et al^[7]^ reported that the central pattern generator can contribute to arm swing during locomotion and other natural movements (e.g., lifting a book, using a cell phone, etc).

An increased locomotion speed exerts a wide range of effects on kinematic characteristics, spatiotemporal parameters, and locomotion patterns in various populations.^[9]^ In particular, it induces significant changes in the movement patterns of the upper limb joints. It is a well-known fact that the upper limb range of motion (ROM) increases proportionally with increasing locomotion speed.^[10]^ However, previous studies have primarily focused on clinical populations such as older adults or patients with Parkinson disease,^[11–13]^ and, from a biomechanical perspective, have been limited to shoulder joint ROM.^[7,14]^ Despite the fact that a considerable body of research has been dedicated to investigating the relationship between lower extremity kinematics and gait speed in various clinical populations,^[15,16]^ there remains a paucity of research investigating comprehensive three-dimensional (3D) ROM changes across multiple lower extremity joints in healthy adults. A substantial body of research has hitherto been dedicated to the study of amputees or the comparison of differences based on biometric factors.^[17,18]^ However, comparatively little attention has been paid to the analysis of multiplanar joint coordination patterns during velocity changes.

The objective of this study was to quantitatively analyze 3D kinematic changes in joints in healthy young adults at different speeds: locomotion, fast locomotion, and running. The analysis of these data will provide valuable insights that will inform the development of devices and rehabilitation protocols for gait and upper limb movement training.

## 2. Methods

This cross-sectional observational study of healthy adults analyzed joint kinematic characteristics at various locomotion speeds. The study was conducted in a university laboratory, within a controlled indoor environment. This study was approved by the Ethics Committee of Kyungwoon University Institutional Review Board (approval code: KW-2024-B-8) on October 16, 2024. All participants provided written informed consent prior to enrollment in the study. This research was conducted ethically in accordance with the World Medical Association Declaration of Helsinki.

### 2.1. Participants

Sample size was calculated using G*Power 3.1.9.7 (Heinrich Heine University Düsseldorf). With an effect size of 0.25, a power of 0.8, and a significance level of 0.05, the minimum required sample size was 128. Considering 20% dropout rate, a total of 155 participants were recruited. All participants consented and were fully informed about the purpose and intended uses of the research prior to participation in the study. They were immediately asked to withdraw from the experiment or take a rest with the onset of pain or any other symptoms.

All participants were university students, 190 healthy adults were recruited, and 155 right-handed participants were ultimately selected as participants for the upper limb limitation condition. Health status was assessed through a self-reported medical history questionnaire and confirmation of inclusion/exclusion criteria. The general characteristics of the participants are presented in Table 1. Inclusion and exclusion criteria are as follows:

**Table 1:** General subject characteristics (N = 155).

Variable	Mean ± SD
Age (yr)	22.57 ± 3.77
Sex (male/female)	89/66
Height (cm)	169.73 ± 7.62
Weight (kg)	70.65 ± 13.25

Data are expressed as means ± SD.

SD = standard deviation.

Inclusion criteria: healthy adults aged 20 to 30 years, right-hand dominant, ability to walk independently, and informed consent.

Exclusion criteria: history of neurological disorders, locomotion disorders due to musculoskeletal diseases, lower limb injury within 6 months, pregnancy, and refusal to participate.

### 2.2. Study protocol

This study was conducted and reported according to the Strengthening the Reporting of Observational Studies in Epidemiology guidelines. The study was performed from September 2023 to March 2024. To simulate hemiplegic gait patterns and provide comparative baseline data for future clinical studies, an upper limb restriction condition was implemented. This approach allows comparison of kinematic adaptation strategies between unrestricted and restricted upper limb conditions in healthy adults, which can inform rehabilitation protocols for patients with stroke. Shoulder and elbow joints were selected as primary measurement sites, as these joints contribute most significantly to arm swing dynamics during locomotion.^[7]^ The self-selected overground locomotion speed was set as 100%, fast locomotion as 130%, and running as 150%. Each locomotion speed was repeated 3 times, and the average of the 3 trials was used for statistical analysis to ensure data reliability. To realize the locomotion form of patients with hemiplegia due to brain injury, the shoulder and elbow of the dominant arm were fixed using a sling. An additional locomotion test was carried out by fixing the trunk and arm. Locomotion speed was specified as normal locomotion pace in a 10-m locomotion test. The ROM of the upper and lower limbs and trunk, and locomotion cycle were assessed according to participants’ locomotion speeds using a 3D motion analysis system.

**Figure 1.**
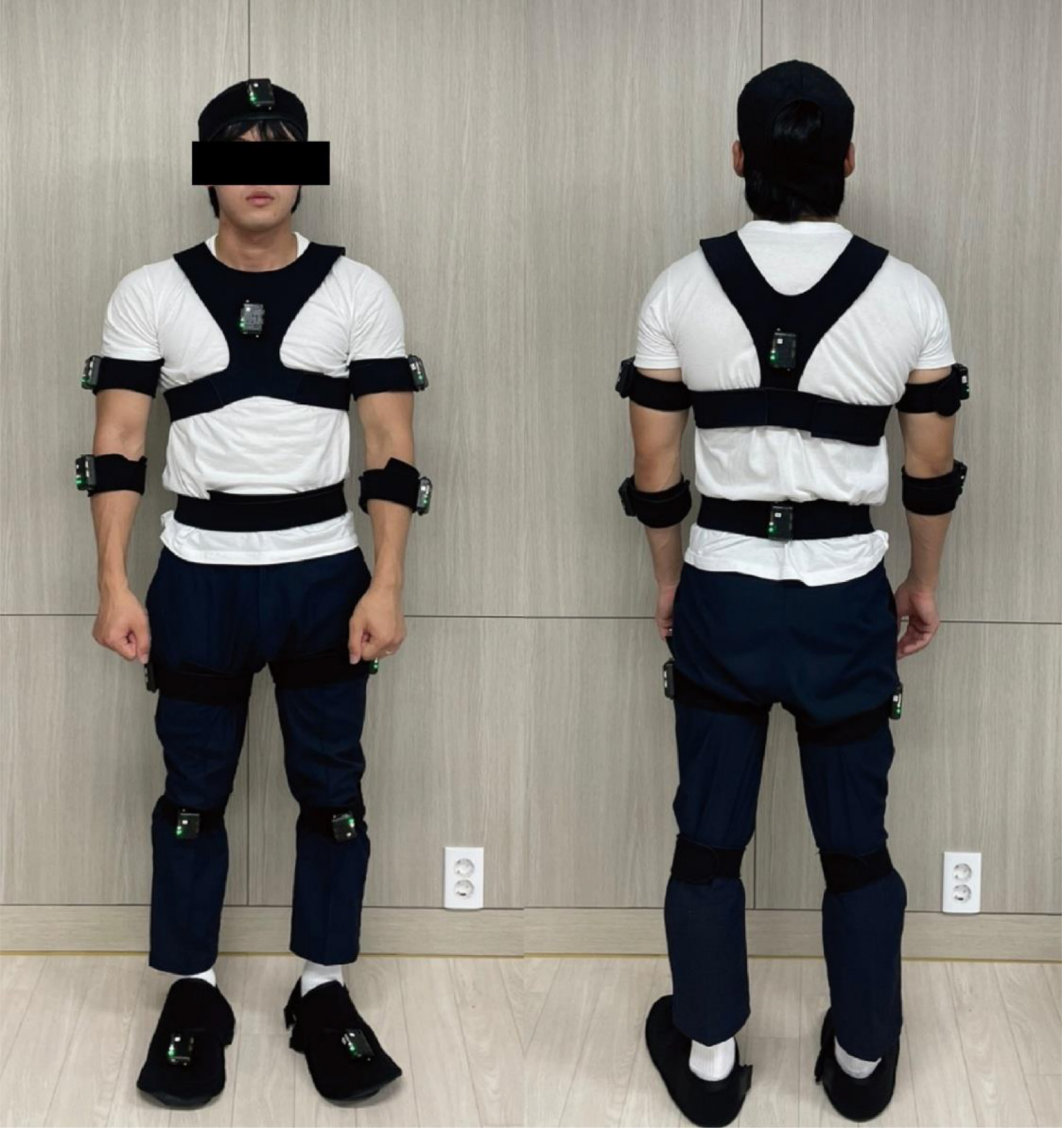
Sensor attachment location.

The experiment was structured similarly to previous studies.^[19]^ To collect and evaluate data on joint ROM during locomotion, iSen (STT-IWS, STT Systems) was used and motions were captured with 16 channels of a 3D motion analysis system. Participants in the study were equipped with 16 inertial sensors following a biomechanical whole-body model (Fig. 1) and attached to the different parts of the body by a semi-elastic Velcro strap. Sensor attachment locations are shown in Figure 1: torso (1), shoulders (2), elbows (2), wrists (2), hips (2), knees (2), ankles (2), and pelvis (1). Each inertial measurement unit sensor (56 × 38 × 18 mm) contains a 3-axis sensor (a magnetometer, accelerometer, and gyroscope) that measure the size of angular motion on 3 axes (x, y, and z). The information from each of these 3 sensors is combined by means of a fusion algorithm (attitude and heading reference system), which allows the calculation of the spatial orientation (3 angles) of the sensor with respect to an absolute reference or with respect to the initial position of the sensor. The use of several sensors placed on different body segments makes it possible to calculate the angles at the joints connecting these segments. The sampling rate was 100 Hz and the connection to the computer was made wirelessly via a standard Wifi network (5 GHz band).^[20]^

### 2.3. Statistical analysis

To explore ROM changes in body joints across various locomotion conditions, statistical analyses were conducted using IBM Statistical Package for the Social Sciences statistics version 28.0 (IBM Corp.) statistics program for Windows. A normal distribution according to the general characteristics of the participants was verified by testing for normality using the Shapiro–Wilk test. Data were expressed as means ± standard deviation. To compare body segment ROM according to locomotion speed, a one-factor repeated measures analysis of variance (ANOVA) was performed. In the event that substantial discrepancies in ROM were identified according to locomotion speed, a post hoc analysis was conducted. The statistical significance level for the main repeated measures ANOVA was set at α = 0.05. For post hoc pairwise comparisons, the Bonferroni correction was applied: α = 0.05/3 = 0.0167 (3 pairwise comparisons: normal vs fast, normal vs running, and fast vs running) to control for Type I error across multiple comparisons.

## 3. Results

A total of 155 healthy adults participated in this study. Demographic characteristics are presented in Table 1 (age: 22.57 ± 3.77 years; 89 males, 66 females; height: 169.73 ± 7.62 cm; weight: 70.65 ± 13.25 kg; Table 1).

A one-way repeated measures ANOVA was employed to compare ROM according to locomotion speed (Table 2). The findings demonstrated that all joint ROM, with the exception of pelvic ROM, exhibited a significant increase with increasing locomotion speed (*P* < .05).

**Table 2:** Comparison of range of motion according to locomotion speed.

Measure		Locomotion velocity	Range of motion	*F*	*P*
Shoulder joint					
Sagittal plane motion	Rt.	Locomotion	26.99 ± 12.97†	76.305	.000*
		Fast locomotion	37.7 ± 17.35		
		Running	42.79 ± 15.36		
	Lt.	Locomotion	31.7 ± 12.26†	64.821	.000*
		Fast locomotion	41.58 ± 17.14		
		Running	47.55 ± 17.31		
Frontal plane motion	Rt.	Locomotion	9.8 ± 5.21†	93.633	.000*
		Fast locomotion	11.95 ± 7.05		
		Running	19.08 ± 8.76		
	Lt.	Locomotion	11.75 ± 6.63†	67.930	.000*
		Fast locomotion	14.77 ± 7.87		
		Running	20.96 ± 10.1		
Transverse plane motion	Rt.	Locomotion	26.54 ± 12.86†	64.337	.000*
		Fast locomotion	34.71 ± 15.38		
		Running	40.14 ± 13.75		
	Lt.	Locomotion	31.07 ± 13.14†	53.811	.000*
		Fast locomotion	38.63 ± 15.92		
		Running	44.37 ± 14.98		
Elbow joint					
Sagittal plane motion	Rt.	Locomotion	21.24 ± 13.4†	79.392	.000*
		Fast locomotion	30.26 ± 15.55		
		Running	48.81 ± 31.81		
	Lt.	Locomotion	26.78 ± 13.98†	130.495	.000*
		Fast locomotion	36.16 ± 15.13		
		Running	27.75 ± 58.54		
Pelvic motion	Tilt	Locomotion	9.41 ± 2.86†	101.223	.000*
		Fast locomotion	4.21 ± 14.27		
		Running	7.9 ± 17.41		
	Obliquity	Locomotion	7.79 ± 2.34†	37.418	.000*
		Fast locomotion	9.26 ± 2.66		
		Running	10.14 ± 3.72		
	Rotation	Locomotion	20.41 ± 8.41	2.197	.11
		Fast locomotion	20.53 ± 6.44		
		Running	21.8 ± 7.28		
Hip sagittal plane motion	Rt.	Locomotion	41.88 ± 7.82	99.671	.000*
		Fast locomotion	52.99 ± 8.2‡		
		Running	52.24 ± 12.45		
	Lt.	Locomotion	43.83 ± 6.66	111.258	.000*
		Fast locomotion	54.3 ± 7.81‡		
		Running	53.48 ± 10.69		
Hip transverse plane motion	Rt.	Locomotion	19.03 ± 4.67	16.678	.000*
		Fast locomotion	22.37 ± 6.21‡		
		Running	20.89 ± 7.36		
	Lt.	Locomotion	16.12 ± 4.83†	16.404	.000*
		Fast locomotion	17.72 ± 4.77		
		Running	20.75 ± 6.43		
Knee sagittal plane motion	Rt.	Locomotion	54.16 ± 7.13§	18.282	.000*
		Fast locomotion	54.32 ± 6.78		
		Running	58.68 ± 13.13		
	Lt.	Locomotion	54.47 ± 9.43§	24.943	.000*
		Fast locomotion	54.35 ± 9.89		
		Running	59.59 ± 12.69		
Ankle sagittal plane motion	Rt.	Locomotion	34.929 ± 13.6	12.298	.000*
		Fast locomotion	38.73 ± 13.27‡		
		Running	40.26 ± 13.48		
	Lt.	Locomotion	38.86 ± 14.19†	40.596	.000*
		Fast locomotion	42.89 ± 12.75		
		Running	47.52 ± 14.64		

† A significant differences among all pairwise comparisons.

‡ No significant difference between fast locomotion and running.

§ No significant difference between locomotion and fast locomotion.

* *P* < .05; mean ± standard deviation.

Subsequent post hoc analyses were conducted on alterations in upper extremity ROM in relation to locomotion speed, unveiling substantial disparities in shoulder and elbow ROM across all dimensions between locomotion, fast locomotion, and running (*P* < .05).

Subsequent post hoc analyses were conducted on alterations in lower extremity ROM in relation to locomotion speed, unveiling substantial disparities in pelvic tilt and oblique ROM, transverse ROM of the left hip, and sagittal ROM of the left ankle among the locomotion, fast locomotion, and running groups (*P* < .05). Furthermore, a significant difference was identified between fast locomotion and running with respect to sagittal ROM of the left and right hip joints, transverse ROM of the right hip joint, and sagittal ROM of the right ankle (*P* < .05). For knee sagittal plane ROM, no significant difference was observed between normal locomotion and fast locomotion (*P* > .05). However, significant differences were found between normal locomotion and running (*P* < .05) and between fast locomotion and running (*P* < .05).

## 4. Discussion

The objective of this study was to quantitatively analyze 3D ROM changes in upper and lower limb joints according to varying locomotion speeds in healthy adults. This was achieved by examining changes in joint ROM according to locomotion speed in healthy participants.

The findings of the study demonstrated that the ROM of the shoulder and elbow joints exhibited a significant increase in proportion to the increase in locomotion speed. Furthermore, the post hoc analysis revealed that this ROM also increased significantly as locomotion speed increased. This finding aligns with the conclusions of earlier research, which demonstrated that the movement pattern of the upper limb undergoes alterations in conjunction with an increase in locomotion speed.^[10]^ Dignwell et al^[21]^ reported that older adult participants and patients with locomotion disorders exhibited a significantly slower locomotion speed than the healthy control group. This was identified as a locomotion strategy employed for dynamic stability control during locomotion. However, Matuszewska et al^[22]^ reported that a slow locomotion speed strategy was not the optimal strategy for locomotion stability through an analysis of locomotion posture according to age, and that symmetrical arm swing was a major factor in stability. Furthermore, Hejrati et al^[23]^ reported that unstable environments, such as various speeds, slopes, and ground conditions during locomotion, significantly affect the size and pattern of arm swing. The results of this study are consistent with those of previous studies, which indicate that increased upper limb joint ROM is associated with increased speed. Increased locomotion speed may be employed as a locomotion strategy, necessitating greater upper limb ROM and coordination. Moreover, increased upper limb ROM is not merely a means of ensuring dynamic stability; it also plays a significant role in locomotion efficiency. It has been demonstrated that arm swing during locomotion reduces energy consumption by offsetting the angular momentum generated by the lower limbs and suppresses excessive trunk rotation, thereby contributing to improved locomotion stability.^[24,25]^ Consequently, the augmented upper limb ROM evident in this study at elevated speeds is regarded as an active strategy for attaining both locomotion stability and energy efficiency. Moreover, the findings of this study imply that the ROM of the upper limbs, lower limbs, and trunk do not merely change individually, but rather function complementarily as locomotion speed increases. In summary, the augmentation of the upper limb’s ROM can be interpreted as a meticulously orchestrated strategy that serves to uphold trunk stability. This is achieved by meticulously balancing the escalating propulsive power of the lower limbs, thereby underscoring the profound interconnection between locomotion and the orchestrated movement pattern of the entire body. Plotnik et al^[26]^ reported that the bilateral coordination that occurs during locomotion becomes more stable as locomotion speed increases. Furthermore, Meyns et al^[7]^ reported that the central pattern generator on both sides of the spinal cord are mutually activated and maintained, contributing to coordination with the lower limbs through the activation of arm muscles during locomotion. In consideration of the findings from preceding studies, the observed complementary coordination among the upper limbs, lower limbs, and trunk in this study is presumably attributable to cross-modal motor pattern regulation at the neural level.

Although the right upper limb was fixed in a sling during the restriction condition, minimal ROM changes were observed in the shoulder and elbow joints (Table 2). This finding likely reflects: residual movements within the sling constraint, particularly shoulder girdle compensations; high sensor sensitivity detecting small movements (100 Hz sampling); and inherent measurement variability. Future studies should employ more rigid fixation methods or validate restriction effectiveness through pilot testing to ensure complete immobilization if intended.

In this study, the majority of lower extremity joints demonstrated increased ROM in response to increased locomotion speed. The hip, knee, and ankle joints exhibit increased ROM, which is likely to facilitate practical mobility and propulsion. The act of locomotion is achieved through interaction with the ground, and the ground reaction force generated when the foot makes contact with the ground can be transmitted to the upper body during weight transfer, potentially hindering body weight shift.^[27]^ Furthermore, an increase in locomotion speed is proportional to the propulsive force for horizontal weight shift, which, along with the ground reaction force, increases dynamic instability and requires adjustment. In this context, an increased ROM in the lower extremity joints acts as a compensatory strategy pattern that enables more stable propulsion and deceleration.^[28]^ Therefore, an increased locomotion speed can be interpreted as a greater influence on the magnitude and direction of the ground reaction force, leading to an expansion in the ROM of the lower extremity joints in response. Moreover, a meta-analysis by Fukuch et al^[9]^ reported that as locomotion speed increases, the minimum and maximum joint angles in the human body also increase. The findings of this study lend support to the increased lower extremity joint ROM that was previously observed. Specifically, the horizontal propulsive force generated by increased locomotion speed significantly outweighed the effects of gravity on the body during locomotion. This scenario necessitated a substantial angular compensation to ensure adequate propulsive and braking power.^[29]^

The lack of significant differences between fast walking and running in some lower limb joints may be attributed to the biomechanical transition zone between these 2 gait modes.^[30]^ As speed increases beyond a certain threshold, the gait pattern shifts from a walking pattern (with a double support phase) to a running pattern (with a flight phase), which may involve different coordinative strategies rather than simply increased ROM.

Concurrently, the present study revealed no substantial variance in pelvic ROM in the transverse plane with increasing locomotion speed. As has been previously demonstrated by a number of studies, transverse pelvic ROM is closely related to trunk ROM. It has been identified as a locomotion strategy when locomotion on unstable surfaces, when adopting longer strides, or when carrying heavy objects.^[31–33]^ Nevertheless, in the present study, only 1 condition that differed in terms of locomotion speed was implemented, with no requirement for alterations in direction or task performance. This finding indicates that no substantial disparities in transverse pelvic ROM were identified. This finding indicates that alterations in speed do not necessarily result in concomitant changes in pelvic movement strategy. Instead, the context in which pelvic ROM is manifested in locomotion strategy is influenced by factors other than straightforward changes in speed. It is recommended that future research incorporate additional conditions, such as changes in support surface, and tasks such as carrying objects, with a view to conducting a qualitative analysis of physical strategies during locomotion.

The present study analyzed the 3D kinematic characteristics of the upper and lower extremity joints in healthy adults according to varying locomotion speeds, with a view to elucidating the effect of increased locomotion speed on upper extremity ROM. The results demonstrated that shoulder and elbow ROM increased in proportion to locomotion speed, thus indicating that upper extremity ROM is not merely a passive response but rather an active strategy for maintaining locomotion efficiency and stability. In addition, the ROM of the hip, knee, and ankle joints in the lower extremities also increased with increasing locomotion speed. These results can be interpreted as joint adaptation mechanisms for securing propulsive and braking power. Conversely, no significant changes were observed in transverse pelvic rotation, likely due to the limitations of the experimental conditions, which only varied locomotion speed.

This study has several limitations. First, the sample was limited to healthy young adults (20–30 years), restricting generalizability. Second, only flat-surface walking in controlled conditions was examined. Third, sling fixation may not fully replicate hemiplegic gait, with residual movements detected. Fourth, wrist kinematics and inter-joint coordination were not assessed. Fifth, only ROM was measured, excluding velocity and acceleration. Future studies should include diverse populations, varied environmental conditions, and comprehensive kinematic measurements.

In summary, this study provided experimental evidence for the hypothesis that increased locomotion speed induces significant changes in the kinematic coordination of the upper and lower extremities. This finding underscores the significance of evaluating and training upper extremity ROM during locomotion rehabilitation, thereby pointing to potential clinical applications, including the development of assistive devices to augment upper extremity ROM. However, the present study is limited in scope, in that it only manipulates changes in locomotion speed. It is recommended that future research incorporate a range of environmental variables, including ground conditions and task performance, in order to facilitate a more comprehensive examination of upper and lower extremity coordination strategies.

## Acknowledgments

During the preparation of this manuscript, the authors used Claude (Anthropic) for proofreading and reference formatting. After using this tool, the authors reviewed and edited the content as needed and take full responsibility for the content of the publication.

## Author contributions

**Data curation:** Nam-Jin Jung.

**Funding acquisition:** Nam-Jin Jung.

**Methodology:** Nam-Jin Jung, MinBong Kang.

**Project administration:** Nam-Jin Jung.

**Software:** Nam-Jin Jung, MinBong Kang.

**Conceptualization:** MinBong Kang.

**Formal analysis:** MinBong Kang.

**Investigation:** MinBong Kang.

**Resources:** MinBong Kang.

**Supervision:** MinBong Kang.

**Validation:** MinBong Kang.

**Visualization:** MinBong Kang.

**Writing – original draft:** MinBong Kang.

**Writing – review & editing:** MinBong Kang.
